# Validation of Reference Genes Aiming Accurate Normalization of qRT-PCR Data in *Dendrocalamus latiflorus* Munro

**DOI:** 10.1371/journal.pone.0087417

**Published:** 2014-02-03

**Authors:** Mingying Liu, Jing Jiang, Xiaojiao Han, Guirong Qiao, Renying Zhuo

**Affiliations:** 1 State Key Laboratory of Tree Genetics and Breeding, Chinese Academy of Forestry, Beijing, People’s Republic of China; 2 The Research Institute of Subtropical Forestry, Chinese Academy of Forestry, Fuyang, People’s Republic of China; Southern Illinois University School of Medicine, United States of America

## Abstract

**Background:**

*Dendrocalamus latiflorus* Munro distributes widely in subtropical areas and plays vital roles as valuable natural resources. The transcriptome sequencing for *D. latiflorus* Munro has been performed and numerous genes especially those predicted to be unique to *D. latiflorus* Munro were revealed. qRT-PCR has become a feasible approach to uncover gene expression profiling, and the accuracy and reliability of the results obtained depends upon the proper selection of stable reference genes for accurate normalization. Therefore, a set of suitable internal controls should be validated for *D. latiflorus* Munro.

**Results:**

In this report, twelve candidate reference genes were selected and the assessment of gene expression stability was performed in ten tissue samples and four leaf samples from seedlings and anther-regenerated plants of different ploidy. The PCR amplification efficiency was estimated, and the candidate genes were ranked according to their expression stability using three software packages: geNorm, NormFinder and Bestkeeper. *GAPDH* and *EF1α* were characterized to be the most stable genes among different tissues or in all the sample pools, while *CYP* showed low expression stability. *RPL3* had the optimal performance among four leaf samples. The application of verified reference genes was illustrated by analyzing *ferritin* and *laccase* expression profiles among different experimental sets. The analysis revealed the biological variation in *ferritin* and *laccase* transcript expression among the tissues studied and the individual plants.

**Conclusions:**

geNorm, NormFinder, and BestKeeper analyses recommended different suitable reference gene(s) for normalization according to the experimental sets. *GAPDH* and *EF1α* had the highest expression stability across different tissues and *RPL3* for the other sample set. This study emphasizes the importance of validating superior reference genes for qRT-PCR analysis to accurately normalize gene expression of *D. latiflorus* Munro.

## Introduction

Bamboos (Bambusoideae), a unique member of the monophyletic BEP clade (Bambusoideae, Ehrhartoideae, Pooideae) in grass family (Poaceae), comprise various woody and herbaceous varieties [1]. Woody bamboos are arborescent and perennial plants characterized by remarkable sizes, woodiness and rather striking life history, which also endow it with considerable economic and environmental significance [2]. As one of the most important components of tropical and subtropical forest ecosystems, especially in Asia, they also have occupied an irreplaceable position in the human productive activities and daily life [3]. Approximately a total of 1,500 commercial applications of bamboo have been identified and recorded [3]. In the light of economic and environmental impacts, *Bambusa* and *Dendrocalamus* are regarded as the two most important pachymorph rhizome genera [4]. *Dendrocalamus latiflorus* Munro (*D. latiflorus* Munro), an evergreen species locally known as ‘tropical giant bamboo’, distributes widely in southern China and southeast Asia and plays vital roles as valuable natural resources [5]. However, these precious resources, as well as other bamboos, on which animals and human communities closely depend, are now threatened, since up to half of the woody bamboo species in the world are in danger of extinction which is aggravated by two reproductive traits: semelparity and mast flowering with long intermast periods [5]–[9]. Therefore, it is crucial to determine and characterize the genetic pathways and specific genes involved in bamboo growth, development and flowering.

Measurement of gene expression is of great importance to illustrate the roles of the selected genes in cellular and physiological processes and reverse transcriptase quantitative real-time polymerase chain reaction (qRT-PCR) is considered to be an efficient, sensitive and reliable technique to achieve this goal [10], [11] because of its advantages in sensitivity, specificity, reproducibility, no post-PCR processing and broad dynamic range [12]. As a straightforward approach comparing the abundance of RNAs, the final results would be influenced by many experimental variations such as the quality of RNAs, the quantity of the first-strand cDNA, the specificity of primers, and retro-transcription efficiencies [13], [14]. Therefore, selection of an appropriate normalization strategy is vital for the obtainment of more accurate and biologically meaningful results. The most commonly used approach for normalization is the introduction of reference genes [10], [15]–[17], which are responsible for normalizing expression level of target gene and controlling the possible errors generated during the experiment since the reference genes are handled under the same conditions as the target genes. Hence, the stability of the selected reference genes counts a lot for the acquisition of ideal and reproducible results.

Generally speaking, it is often the genes that are constitutively expressed and play roles in the maintenance of basic cellular function to be chosen as reference genes such as *actin* (*ACT*), *alpha-Tubulin* (*TUA*), *glyceraldehyde-3-phosphate dehydrogenase* (*GAPDH*), *polyubiquitin* (*UBQ*), and *elongation factor 1-α* (*EF1α*) [18]–[25]. However, the selection of the reference genes may vary from species to species or even the same plant under different conditions or at different developmental stages, hence in order to achieve better results, the appropriate reference genes aiming at a specific species need to be assessed.

In recent years, numerous reports on the reference gene validation have been published, such as *Arabidopsis thaliana* [26], rice [27], [28], *Brachypodium distachyon* [29], wheat [30], barley [31], soybean [32], [33], tomato [34], potato [35], sugarcane [36] and poplar [37]. A great deal of genomic or expressional data have been made available for the majority of grasses and many reference genes have been validated to serve for the further studies at the transcriptomic level. However, for bamboo, there is still a long way to go. Although biochemical, physiological and cytogenetic studies of bamboo have been carried out for several decades, factors such as the deficiency of the genomic resources would hamper biological investigations of this group of morphologically and physiologically unique and ecologically and economically important grasses [38]. In our previous research, the transcriptome sequencing for *D. latiflorus* Munro has been performed [39]. Numerous genes involved in growth and development, especially those predicted to be unique to *D. latiflorus* Munro, were revealed. How about their spatial and temporal expression profiles? How about their responses to biotic and abiotic stresses? To solve these issues, a set of suitable internal controls should be validated for *D. latiflorus* Munro to identify the transcription and expression of genes interested and accelerate the genetic studies of bamboo.

In this report, twelve candidate reference genes were chosen for the assessment of the expression stability, including some housekeeping genes whose stability has been analyzed in other plants and are commonly employed as references in gene expression studies, such as *ACT*, *GAPDH*, *TUA, EF1α*, *Transcription elongation factor* (*TEF*), and *ribosomal RNA 18s* (*18S*). In addition to the genes mentioned above, *ubiquitin-conjugating enzyme* (*UBC*), *peptidylprolyl cis-tans isomerase/cyclophilin* (*CYP*), *ribosomal protein L3* (*RPL3*) and *NAC domain-containing protein* (*NAC*), whose expressions were reported to be stable [40], [41], were also included as candidate reference genes for *D. latiflorus* Munro. Moreover, two genes, *tonoplast intrinsic protein 41* (*TIP41*) and *nucleotide tract-binding protein* (*NTB*), were added in this study as they were found to be homogeneously expressed in *Phyllostachy edulis* (*P. edulis*, Moso bamboo) [42]. This work will contribute to provide more accurate and biologically meaningful results by providing a reliable mean of normalization during qRT-PCR analysis and benefit future studies on gene expression profiles in *D. latiflorus* Munro.

## Results

### Verification of Amplicons, Primer Specificity, Ct Data Collection and Gene-specific PCR Amplification Efficiency

**Figure 1:**
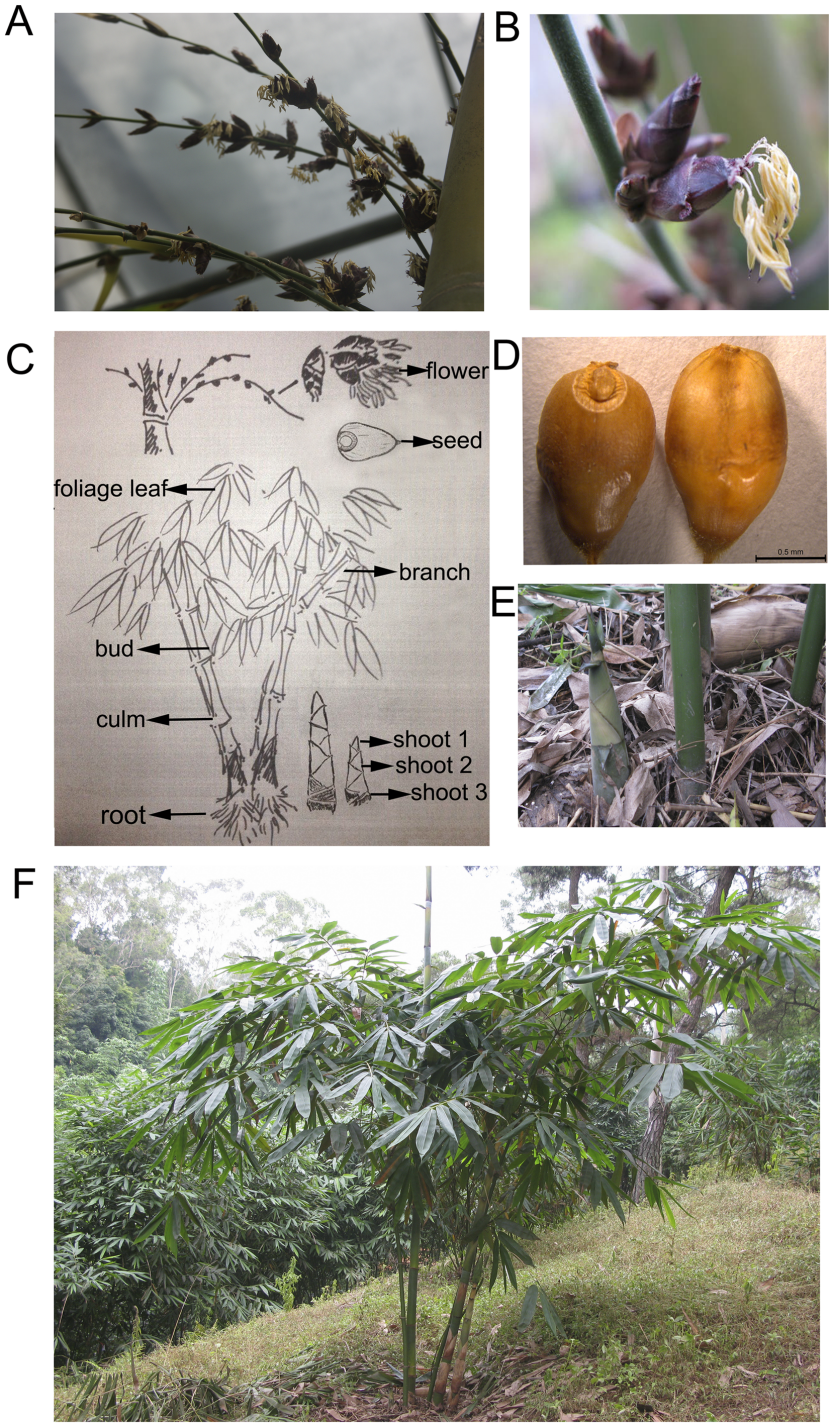
Schematic diagram illustrating different tissue origins used for qRT-PCR and photos represent specific tissues. (A) and (B) represent the close up of *D. latiflorus* Munro flowers. (C) Schematic diagram illustrating different tissues origin used for qRT-PCR. (D) and (E) represent *D. latiflorus* Munro seeds and shoots. (F) Growth behavior of *D. latiflorus* Munro.

We chose ten representative tissue sample sets of *D. latiflorus* Munro used for qRT-PCR and the schematic diagram of the overall plant and photos displaying some specific tissues were shown in Fig. 1. Moreover, we also collected leaves from three-year-old seedlings and anther-regenerated plants of different ploidy grown in greenhouse to broaden the research scope. In all, ten reference genes were selected from *D. latiflorus* Munro transcriptome dataset and two other genes were attained using homology cloning techniques. The twelve genes mentioned above served as candidates for normalization of gene expression measures and their detailed descriptions are listed on Table 1, including the corresponding accession numbers, gene description and main functions. qRT-PCR was applied to determine the stability of gene expression by quantifying the mRNA level. Primer sequences, amplicon length and amplification efficiency were listed in Table 2.

**Table 1:** Description of twelve candidate reference genes for RT-qPCR in *D. latiflorus* Munro.

Gene Symbol	Accession number	Gene Description	Gene Function
***ACT***	KF467245	*Actin 2*	Essential components of cytoskeleton
***GAPDH***	KF484750	*Glyceraldehyde-3-phosphate dehydrogenase*	Catalyzing energy-yielding step in carbohydrate metabolism
***NAC***	KF484751	*NAC domain-containing protein*	Sequence-specific DNA binding transcription factor
***TUA***	KF484754	*Tubulin alpha-3*	Major components of microtubules
***UBC***	KF484755	*ubiquitin-conjugating enzyme*	Ubiquitin-protein ligase activity
***EF1α***	KF484749	*Elongation factor 1-alpha*	Enzymatic delivery of aminoacyl tRNAs to the ribosome
***CYP***	KF484748	*Peptidyl-prolyl cis-trans isomerase/cyclophilin*	Playing roles in the isomerization of peptide bonds
***RPL3***	KF484752	*ribosomal protein L3*	Structural constituent of ribosome 60S subunit
***18S***	KF467246	*18s ribosomal RNA*	Structural RNA for the small cytosolic ribosomes
***TEF***	KF484753	*Transcription elongation factor*	Regulating the transcription of protein-coding genes
***TIP41***	KF765496	*tonoplast intrinsic protein 41*	Working as water channel
***NTB***	KF765497	*nucleotide tract-binding protein*	an element of a compound necessary for pre-mRNA splicing

**Table 2:** Selected candidate reference genes and target genes, primers and different parameters derived from qRT-PCR analysis.

Gene Symbol	Primer pair (forward/reverse)	Product Size (bp)	R^2^ *	PCR efficiency
***ACT***	TGCTCTCCCCCATGCTATCCTTC/ATGTCCCTCACAATTTCCCGCTC	130	0.9991	100.09%
***GAPDH***	CCGTCAACGACCCCTTCATCACC/TACTCAGCACCAGCCTCAGCCCA	193	0.9992	99.38%
***NAC***	TGATGCTGTGGCTCCTAAGGTTGAA/TAGCAAAAGCAAGAGCTGCCTGATC	173	0.9991	100.25%
***TUA***	GTTGGTGGTGGAACTGGGTCAGG/TCAAGCAAGGAGTGGGTGGAGAG	167	0.9980	96.74%
***UBC***	CCCAGCCCTTACCTTGAAGACGG/TAACGAGCAGTGGCAACAAATGTGG	135	0.9980	97.61%
***EF1α***	GTTGTTACCTTTGGTCCCAGCGG/TGGCAGGGTCATCCTTGGAGTTG	175	0.9984	98.55%
***CYP***	GGTGGTGAACCCAAAAATGCTGCTAC/ACCCTTTCTTCCCATTCTCATTATCTGC	172	0.9986	99.76%
***RPL3***	AGGAGTAGGCAGGGGAAGATGTCGC/TTGTAGCCAAGGAAGGCAGTGAGGTG	167	0.9976	96.93%
***18S***	ATGCTTGGCAAATGCTTTCGCTGTA/ATGATTAAGAGGGACTGACGGGGGC	101	0.9984	99.48%
***TEF***	CTCTTCGCCACGCCAACCACTTC/GTCGTCCACCGTCCTTGCCACC	120	0.9978	98.82%
***TIP41***	TTGGTTTCTGCTCTTGCGTTTTTGG/ACGGGTTTTGCTTCCTCATTATTTCC	106	0.9991	97.69%
***NTB***	TTGCTGTGCTCCAACCAAGATGA/TCAAACAAGACGAGAGCCTGCCT	153	0.9979	98.32%
***Ferritin***	TACTTTCGGCTGCGCCATGT/CGGGATGCTGAAGGAAAT	188	0.9980	98.66%
***Laccase***	TGGAAGGTGACGTTGTTGTT/TCAATGTGACAATGGCGACT	153	0.9989	97.47%

* R^2^ represents the correlation coefficients.

**Figure 2:**
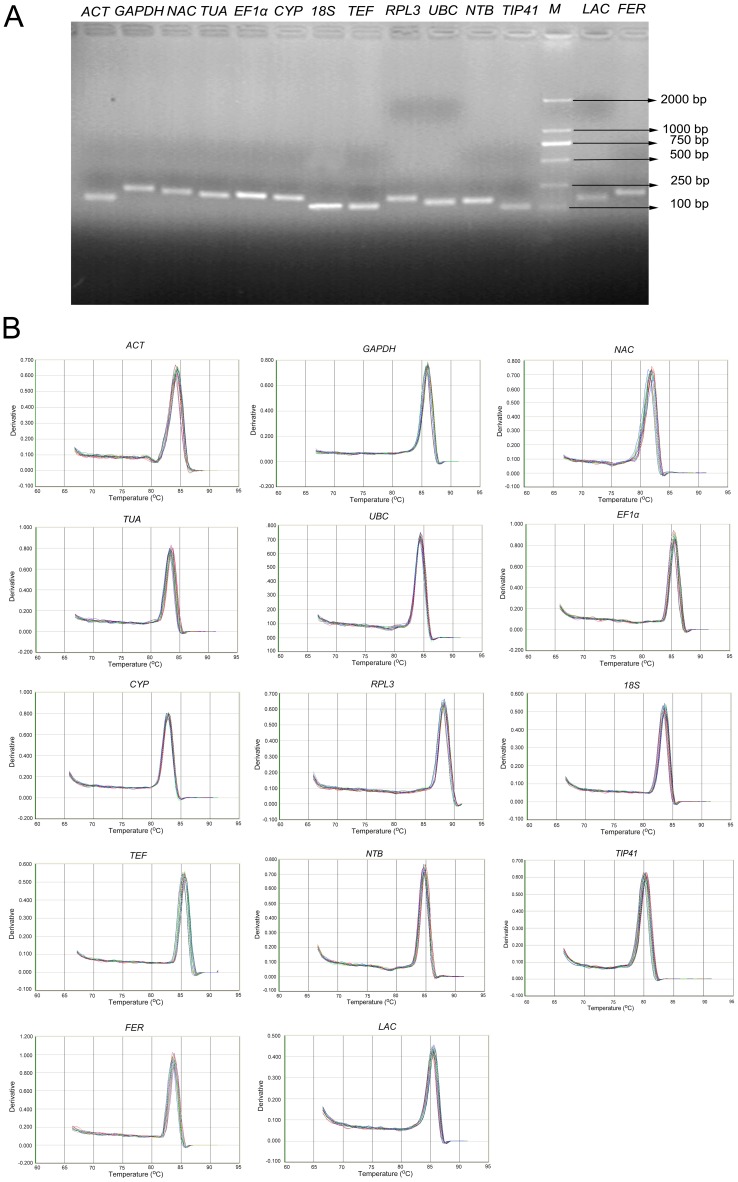
Identification of gene specificity and amplicon size. (A) Agarose gel (2%) electrophoresis showing amplification of a specific PCR product of the expected size for each gene. (B) Melting curves of twelve candidate reference genes and two target genes showing single peaks.

Agarose gel electrophoresis was performed to test for the specificity of the amplicons. Melting curve analysis was conducted to verify the presence of gene-specific peaks and the absence of primer dimers. The melting temperatures (Tm) of all PCR products ranged from 80.4°C for *18S* to 88.3°C for *RPL3*. Amplification efficiency (E) of PCR reactions varied from 96.74% for *TUA* to 100.25% for *NAC*, and correlation coefficients (R^2^) ranged between 0.9976 for *RPL3* and 0.9992 for *GAPDH*, respectively (Table 2). The specificity of the amplification was confirmed by the single band of expected size for each primer pairs in 2% agarose gel electrophoresis without primer dimers or other PCR products generated from non-specific amplification (Fig. 2A). The single-peak melting curves of the PCR products (Fig. 2B) further indicated that the specificity of the selected primers.

**Figure 3:**
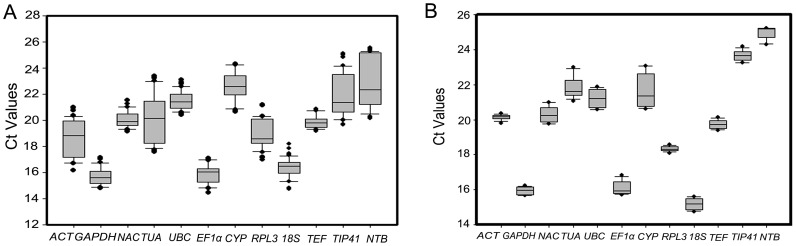
Ct values for twelve candidate reference genes in qRT-PCR. (A) Expression levels of candidate genes across different tissues of *D. latiflorus* Munro. (B) Expression levels of candidate genes among samples from seedlings and anther-regenerated plants of different ploidy. The boxes represent mean Ct values and bars corresponding to the standard deviation. The Ct values were calculated on 1∶30 diluted cDNA samples.

The cycle threshold (Ct) value, which is the intersection between an amplification curve and a threshold line, was calculated for each gene. It is generally characterized by the middle of exponential phase of amplification [43]. The Ct range and the coefficient of variance for each gene across all samples could help to reveal the differences in transcript levels among twelve candidate reference genes. When the expression data from ten tissue samples were analyzed, the Ct values of these twelve candidate reference genes varied widely, ranging from 14 to 26 cycles (Fig. 3A), but most of the Ct values were between 17 and 23 cycles. *GAPDH*, *EF1α* and *18S* were characterized by most abundant expression with Ct values of less than 18 cycles and showed narrow variance (Fig. 3A). The Ct values of *TEF* were between 19 and 21 cycles and displayed a small gene expression variation (below 3 cycles) (Fig. 3A). *NTB* and *TIP41* had much higher expression variations with Ct values between 19 and 26 cycles, so did *ACT* and *TUA* whose Ct values were between 16 and 24 cycles (Fig. 3A). In the case of four leaf samples from seedlings and anther-regenerated plants, *GAPDH*, *EF1α* and *18S* were highly expressed compared to others (Fig. 3B). The variances for *ACT* and *RPL3* were the smallest while *CYP* displayed much higher expression variations (Fig. 3B). *NTB* had the least abundance, reaching threshold fluorescence at 24 to 26 amplification cycles (Fig. 3B). *TUA,* which was normally used as the reference gene, exhibited much greater variations in its expression levels in the two experiment sets. According to the analysis, we could find that *GAPDH* and *EF1α* had a relatively constant expression in different samples while some other genes showed different expression variation such as *ACT* and *TUA*. Thus, it is crucial to select reliable reference genes to gain accurate expression data.

### GeNorm Analysis

Gene expression stabilities of the twelve candidate genes in different samples were analyzed by geNorm software as described by Vandesompele et al. [24]. GeNorm automatically calculates the average gene expression stability measure (M) based on the average pair-wise variation between all genes tested and determines the average pairwise variation (V) values with all other control genes as the standard deviation of the logarithmically transformed expression ratios [29]. M value of 1.5 is recommended as the screening criteria and those genes with M value below this threshold are identified as ideal reference genes with stable expression. The gene with the lowest M value is deemed to be the one showing the most stable expression, while the gene with the highest M value is supposed to display the least stable expression.

**Figure 4:**
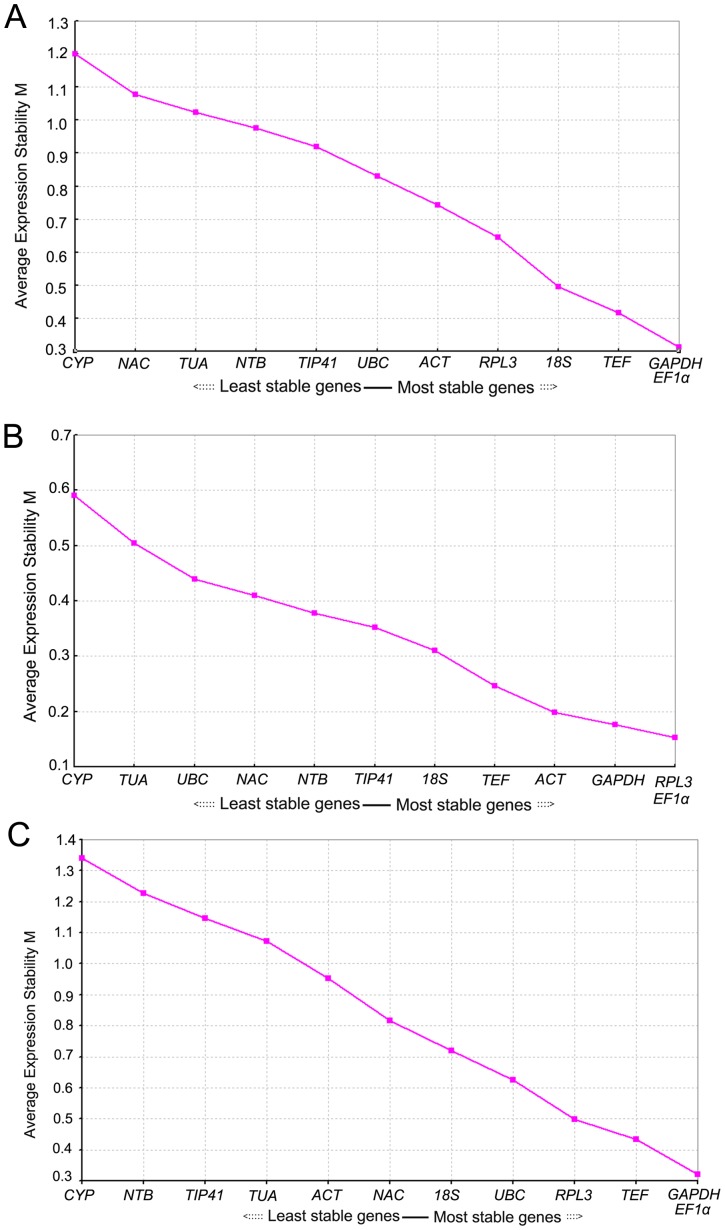
Gene expression stability of the twelve candidate genes of *D. latiflorus* Munro as predicted by geNorm. (A) Different tissues, (B) Different samples from seedlings and anther-regenerated plants of different ploidy, (C) All samples. Lower average expression stability (M value) indicates more stable expression. The least stable genes are on the left, and the most stable on the right.

Our analysis showed that M values of all twelve selected genes were smaller than 1.5, which indicated that all of them conformed to a basic requirement for the reference genes. As shown in Fig. 4A, when the results from ten tissue samples of *D. latiflorus* Munro were analyzed, *EF1α* and *GAPDH* showed the highest expression stability with the lowest M value (0.32) and *CYP* was found to have the highest M value (1.21) being the least stable gene. The M value of *TEF* was 0.415 and it could also be used as a reference gene. In the four leaf samples from seedlings and anther-regenerated plants of different ploidy, the results recommended a different pair of ideal reference genes, *EF1α* and *RPL3*, with the lowest M value (0.152) while *CYP* was still ranked at the bottom (Fig. 4B). *GAPDH* and *EF1α*, although not the top two, were still predicted to be stable genes, occupying the third and fourth place, with the M value around 0.2 (Fig. 4B). When all the samples were taken together, the analysis showed that *GAPDH* and *EF1α* were evaluated to be the most stable genes with high statistical reliability and *TEF* and *RPL3* were ranked the third and fourth (Fig. 4C). The two reference genes identified in Moso bamboo, *TIP41* and *NTB*, did not perform well in *D. latiflorus* Munro. For example, in the case of ten tissue samples, the M value for *TIP41* was 0.912 and the M value for *NTB* was 0.979, being the eighth and ninth respectively. In brief, our analysis manifested that *GAPDH* and *EF1α* could serve as reference genes among ten tissue samples or all samples taken together while *TEF* and *RPL3* were also qualified to be used for standardization. *EF1α* and *RPL3* were evaluated to be the most stable gene across samples from the seedlings and anther-regenerated plants of different ploidy.

Although in general only one single gene was used as an internal control for normalization by most researchers, it has been suggested that the application of two or more reference genes for qRT-PCR studies might generate more reliable results [24], [25], [44]. The optimal number of reference genes was also calculated by the geNorm program for the accurate normalization among the different sample sets. The pair-wise variation V_n_/V_n+1_ was determined as an indicator, which represents the effect of adding further reference genes on the normalization factor (that is calculated as the geometric mean of the expression values of the selected reference genes). It is feasible to add additional reference genes to the normalization factor until the pair-wise variation V_n_/V_n+1_ drops below a given threshold (0.15) recommended by Vandesompele et al. [24], which means the added gene has no significant effect and the inclusion of an additional reference gene is not required.

**Figure 5:**
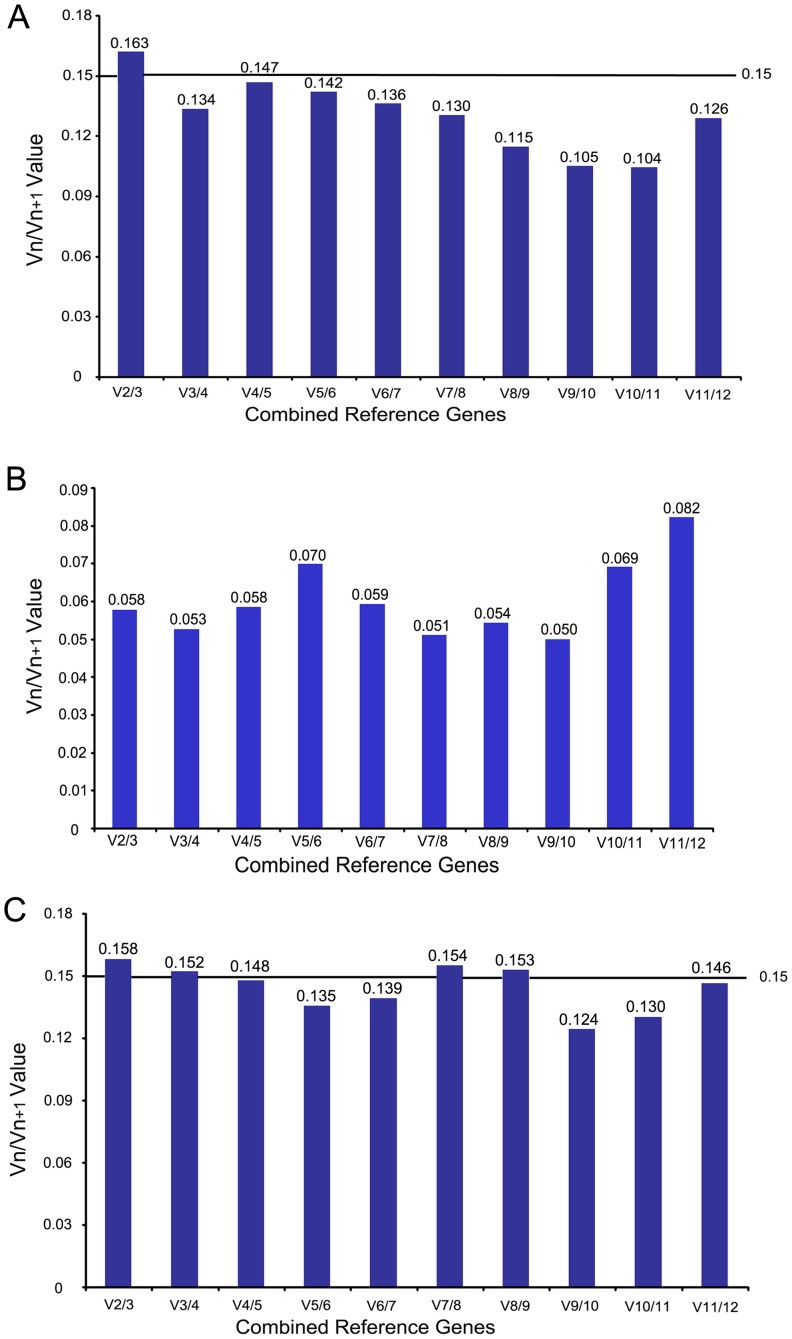
The optimal number of reference genes required for effective normalization across different tissues of *D. latiflorus* Munro. (A) Different tissues, (B) Different samples from seedlings and anther-regenerated plants of different ploidy, (C) All samples. The pairwise variation (Vn/Vn+1) was analyzed between normalization factors NFn and NFn+1 by geNorm program to determine the optimal number of reference genes.

As shown in the Fig. 5A, in ten tissue sample sets, the use of the third reference genes contributed significantly to the variation of the normalization factor since the V3/4 value was under the 0.15 cut-off level. When the samples from seedlings and anther-regenerated plants of different ploidy were tested, all the pair-wise variations dropped below the given threshold (0.15) (Fig. 5B) indicating that one reference gene is adequate for the normalization. When all the samples were taken together, both V2/3 and V3/4 were higher than the cut-off value (0.15), being 0.158 and 0.152 respectively (Fig. 5C). Until V4/5, the value was below the threshold (Fig. 5C), indicating that the addition of the fourth internal gene was necessary to normalize gene expression. Therefore, according to geNorm results, it is essential to assess a set of appropriate reference genes for the more accurate analysis of the further research results.

### NormFinder Analysis

The expression stability of candidate reference genes was further evaluated by NormFinder software, which is based on a variance estimation approach. More stable gene expression is indicated by lower average expression stability values. This program could combine the variation of both intra- and inter-group variation and a single analysis of the sample subgroups in expression levels into the calculation of a gene expression stability value [45]. The expression stability values and the ranking order of different experiment sets evaluated by the Normfinder program were summarized in Table 3.

**Table 3:** Ranking of the candidate reference genes according to their stability value using NormFinder.

Rank	Tissues		Seedlings and different ploidy		Total	
	Gene name	Stability Value	Gene name	Stability Value	Gene name	Stability Value
**1**	*GAPDH*	0.160	*RPL3*	0.029	*EF1α*	0.080
**2**	*EF1α*	0.163	*EF1α*	0.078	*GAPDH*	0.143
**3**	*TEF*	0.369	*TEF*	0.102	*RPL3*	0.345
**4**	*RPL3*	0.423	*18S*	0.114	*TEF*	0.559
**5**	*18S*	0.464	*GAPDH*	0.177	*UBC*	0.619
**6**	*TIP41*	0.602	*ACT*	0.228	*NAC*	0.645
**7**	*UBC*	0.629	*NAC*	0.266	*ACT*	0.704
**8**	*ACT*	0.639	*TIP41*	0.329	*18S*	0.762
**9**	*NTB*	0.681	*NTB*	0.366	*TIP41*	0.817
**10**	*NAC*	0.736	*UBQ*	0.407	*TUA*	0.889
**11**	*TUA*	0.792	*TUA*	0.468	*NTB*	1.055
**12**	*CYP*	1.144	*CYP*	0.678	*CYP*	1.184

NormFinder software ranked *GAPDH* and *EF1α* as the two most stable genes among ten tissue samples (Table 3). *TEF* and *RPL3* were also characterized with relatively low expression stability value (0.369 and 0.423 respectively) and ranked among the top four candidate genes (Table 3). When considering all the experimental samples, the results were broadly similar to the series of different tissues, with the highest stability value for *EF1α* followed by *GAPDH*, *RPL3* and *TEF* with stability values of 0.080, 0.143, 0.345 and 0.559 respectively (Table 3). *RPL3* was calculated to be the most reliable reference gene for the samples collected from seedlings and anther-regenerated plants of different ploidy (Table 3).

*CYP* exhibited unstable expression profile and were always classified as the most variable one in three analyses (Table 3). *TUA*, a commonly used reference gene, was ranked at the bottom in all the three experimental sets, hence was evaluated to be not appropriate as the reference gene (Table 3). *TIP41* ranked the sixth among ten tissue samples, eighth in samples from seedlings and anther-regenerated plants of different ploidy and ninth when all the samples taken together, indicating its instability of expression (Table 3). *NTB* also performed poorly and were included among the least stable reference genes. This result further confirmed that the two ideal reference genes for Moso bamboo were not competent for the validation of gene expression data in *D. latiflorus* Munro and emphasized the necessity of characterizing proper reference genes for a specific species.

The NormFinder analysis revealed a remarkable expression stability of *GAPDH* and *EF1α* in ten tissue sample or all the sample pools with *TEF* and *RPL3* being alternatives, while *RPL3* was the most stable gene among samples from seedlings and anther-regenerated plants of different ploidy, which were in accordance with the results analyzed by geNorm analysis tool.

### Bestkeeper Analysis

BestKeeper is a tool based Excel and it evaluates inter-gene relevance of candidate reference gene pairs through conducting pairwise correlation analysis utilizing raw Ct values. The determination of genes with the most stable expressions was based on the coefficient of correlation (r) to the BestKeeper Index (BI) and those showing a strong correlation with the BestKeeper index were regarded as stable reference genes [46]. Based on the results produced by NormFinder and geNorm analysis, we omitted the two least stable genes in each experiment set and the results calculated by BestKeeper were summarized in Table 4.

**Table 4:** Expression stability values of the candidate reference genes calculated using BestKeeper.

Rank	Tissues		Seedlings and different ploidy		Total	
	Gene name	Coeff. of corr (r)*	Gene name	Coeff. of corr (r)*	Gene name	Coeff. of corr (r)*
**1**	*EF1α*	0.95	*RPL3*	0.97	*EF1α*	0.92
**2**	*GAPDH*	0.93	*TEF*	0.94	*GAPDH*	0.90
**3**	*RPL3*	0.92	*EF1α*	0.93	*RPL3*	0.85
**4**	*TEF*	0.90	*GAPDH*	0.82	*ACT*	0.83
**5**	*ACT*	0.86	*UBC*	0.57	*TEF*	0.76
**6**	*TIP41*	0.86	*NAC*	0.52	*TIP41*	0.76
**7**	*NTB*	0.82	*18S*	0.48	*TUA*	0.68
**8**	*TUA*	0.67	*ACT*	0.35	*NAC*	0.54
**9**	*18S*	0.51	*TIP41*	0.28	*UBC*	0.31
**10**	*UBC*	0.46	*NTB*	0.27	*18S*	0.12

* Coeff. of corr (r) is the shortage of Coefficient of correlation.

The BestKeeper analysis revealed that when ten tissue sample were introduced, the best correlations were obtained for *EF1α* (r = 0.95), *GAPDH* (r = 0.93), *RPL3* (r = 0.92) and *TEF* (r = 0.90) with p value of 0.001 (Table 4). When we evaluated the expression data of samples from seedlings and anther-regenerated plants of different ploidy, *RPL3, TEF* and *EF1α* were ranked as the three top genes showing strong correlation with BestKeeper index (r>0.90) (Table 4). The high Pearson's coefficients of correlation indicated that the expression profiles of the above gene pairs were similar. When considering all the samples, *EF1α* and *GAPDH* had strong correlation with the BestKeeper index (r>0.90) while *RPL3* and *ACT* were in the third and fourth place. *TEF* moved to the fifth with r value being 0.76 (Table 4).

*NTB* and *TIP41* were identified as the most unstable genes in the samples from seedlings and anther-regenerated plants of different ploidy and fell in the bottom in the other two experiment sets (Table 4). *18S* and *UBC* were determined as genes with the least stability when considering all the samples or just ten tissue samples and not proper to be used as the normalization standard (Table 4).

In the three experiment sets, *EF1α* displayed strong correlation with the BestKeeper index (r>0.90), and were ranked among the top four genes, in accordance with the corresponding NormFinder and geNorm results. Thus we recommend *EF1α* as the reference gene of first choice for normalization. Although the rank orders for *GAPDH*, *TEF* and *RPL3* were altered slightly among the three experiment sets, they still performed well according to the analysis and could serve as alternative reference genes.

### Reference Gene Validation

**Figure 6:**
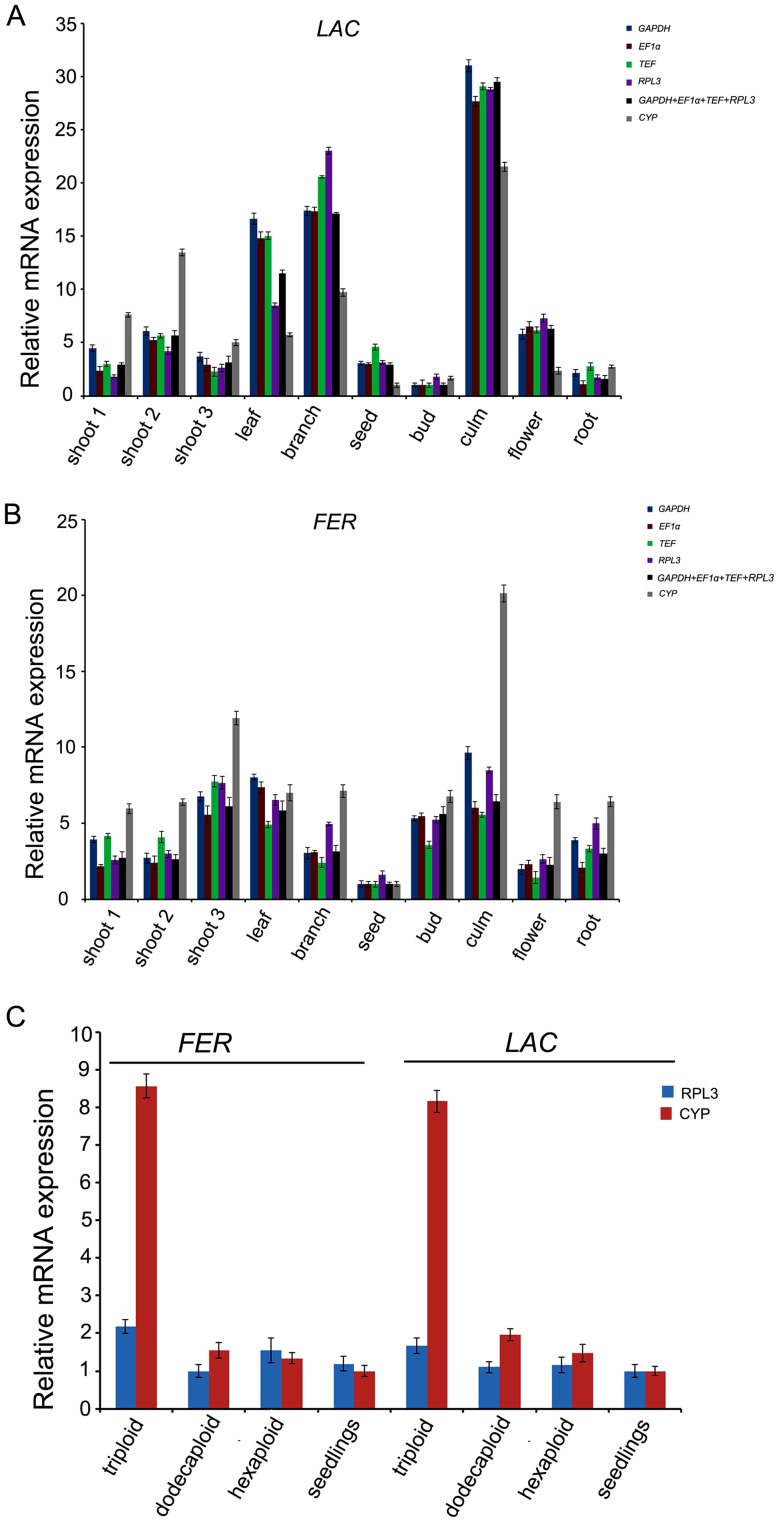
Relative quantification of two target gene expression using identified reference genes for normalization. (A) The expression profile of *laccase* across different tissues of *D. latiflorus* Munro. (B) The expression profile of *ferritin* across different tissues of *D. latiflorus* Munro. (C) The expression profiles of *ferritin* and *laccase* among samples from seedlings and anther-regenerated plants of different ploidy. Genes were normalized to individual and/or combined reference genes. Error bars show the standard error calculated from three biological replicates.

The use of different reference genes to calculate relative expression data could have a significant influence on the final normalized results. To detect the effect of reference gene on the outcome of a practical experiment and further validate the reliability of the recommended reference genes, the relative expression patterns for two functional genes *ferritin* (*FER*) and *laccase* (*LAC*) were evaluated using different reference genes (*EF1α*, *GAPDH*, *TEF*, *RPL3*) and their combination in ten tissue sample sets (Fig. 6A and B). The biological variations of *FER* and *LAC* among seedlings and anther-regenerated plants of different ploidy were characterized by *RPL3* as internal gene (Fig. 6C). As a comparison, the most unstable gene (*CYP*) was also applied for normalization in the analysis.

The lignin content of bamboo is higher than most herbaceous plants and *laccase* is a key member involved in lignin biosynthesis. *Ferritin* is a ubiquitous intracellular protein storing iron and releasing it in a controlled fashion and it is very likely that plant ferritins, by buffering iron, exert an appropriate control of the quantity of metal required for metabolic purposes [47]. According to the results normalized by four top-ranked reference genes (*EF1α, GAPDH, TEF and RPL3*), *LAC* displayed analogical expression characteristics with certain changes in individual samples, being found to be most abundantly expressed in culm and also rich in branch and leaf (Fig. 6A). The results also indicated that *LAC* was shown the lowest expression abundance in the tissue of bud (Fig. 6A). Likewise, the expression diversity of *ferritin* through *GAPDH, TEF, RPL3* or *EF1α* normalization exhibited consistency among different tissue sample sets. *FER* was found abundantly expressed in leaf, shoot 3 and culm. The lowest level of expression was in seed (Fig. 6B). Normalizing the expression data of two target genes by the combination of the above four reference genes provided a consistent expression profile, proving the reliability of the recommended reference genes. Moreover, it could be found that the least stable candidate gene *CYP* failed to effectively standardize the expression data as it over- or underestimated the target gene abundance such as the *FER* expression in culm or the *LAC* expression in shoot 2. In a word, the expression profiles of the two target genes obtained using four reference genes showed a similar trend among different tissues, further demonstrating that a series of recommended genes (*GAPDH, EF1α, RPL3* and *TEF*) could serve as internal genes.

Next, we evaluated expression data of two target genes by *RPL3* with the highest stability and *CYP* with the lowest stability among samples from seedlings and anther-regenerated plants of different ploidy. The analysis showed that the normalization by *CYP* obviously overestimated the expression level of the two target genes in triploid plants (Fig. 6C). The results displayed the adverse impact of adopting an inappropriate reference gene for normalization and further confirmed the importance of validating reference gene stability to improve the accuracy and avoid misleading results.

## Discussion

Due to the sensitivity, accuracy and operability, qRT-PCR has become a feasible approach to relatively quantify the transcript expression levels and uncover the gene expression profiling [48]–[50]. Several factors could exert influences on the quantification of gene expression, for instance, the variations produced from experimental sources and the normalization method. The experimental sources of variation can be sample-to-sample variation in RNA quality, or the different efficiency of reverse transcriptase reaction, or the amount of cDNA template used in each PCR reaction. Application of stable reference genes could normalize the expression level of a target gene to obtain relative quantification across samples and compensate for the variations caused in the experimental and analytical procedures [46]. Hence, the ideal reference gene should maintain invariable expression levels among different cell types, tissues, organs, developmental stages or treatments that are submitted to the tested organism [24], [45]. Therefore, the selection of one, or preferably more, reference genes that are stably expressed across different samples could improve the accuracy and reliability of the results obtained by qRT-PCR [18], [24]. Moreover, correct and accurate sample normalization could help to detect small but significant differences in expression when conducting comparisons among different organs or tissues.

In many plant species, some housekeeping genes, such as *ACT*, ribosomal genes, *ubiquitin 10* (*UBQ10*), *GAPDH*, *EF* and *alpha-tubulin* (*TUA*), are applied as reference genes for gene expression studies [51]–[54]. However, numerous studies have shown that the expression of the so-called ‘housekeeping’ genes, although constant under some experimental conditions, can vary quite considerably in other cases, implying that the expression stability of the intended control gene has to be verified before each experiment [55]–[59]. Moreover, the traditional housekeeping genes validated in one species are not always appropriate for other species [32], [60], [61]. The expression level of some common internal genes also fluctuates under different treatments or experimental conditions. For example, Nicot and colleagues indicated that *ACT* did not serve as the best reference gene as it varied upon different stress treatment [35]. The *actin* gene also showed variable expression in soybean root galls exposed to nematode parasitism and insect attack, suggesting that *actin* is not appropriate reference gene for normalization in conditions of abiotic and biotic stresses [59]. A study conducted by Chen and colleagues showed that the widely used reference genes, *ACT* and *GAPDH*, were not the most suitable reference genes in many banana sample sets [71]. In addition, Gutierrez and colleagues have found some common reference genes, including *ACT*, *TUA*, *UBQ* and *EF*, displayed high variability in the relative expression during various developmental stages in *Arabidopsis* [55]. A study on flower-color gene expression in an azalea (*Rhododendron simsii* hybrids) mapping population also strongly suggests that a thorough assessment of reference genes for expression stability is vital for an accurate quantification [62]. Therefore, the most stable reference gene(s) should be identified for a specific species under study or in a new experimental set-up.

Although *D. latiflorus* Munro is an important bamboo species with great economic and ecological significance, limited molecular research on its growth, development and flowering has already been performed [63]–[65]. Our previous study concerning the transcriptome sequencing for *D. latiflorus* Munro has been completed [39] and numerous unigenes identified were annotated according to their putative functional categories. These sequence and putative function data serve as a valuable resource for future investigation of the growth and development in bamboo species and it will be essential to validate the functions carried out by these genes including discovering their expression profiles. qRT-PCR, which is a rapid, accurate and sensitive technique for relative quantification of transcript expression levels, has become an important tool for accurate gene expression profiling in addition to Northern blotting [48]–[50]. Accurate normalization is an absolute prerequisite for correct measurement of gene expression, and the strategy most commonly applied for normalization is standardization to a constitutively expressed internal gene. Therefore, it is of great significance to perform the study aiming validation of ideal reference genes for accurate normalization of qRT-PCR data. It has been strongly suggested that the most stable reference gene(s) should be identified for a specific species under study or in a new experimental set-up [66]. Hence, suitable reference genes for *D. latiflorus* Munro needs to be established in advance to the experiments accordingly.

We thus chose ten candidate reference genes for which sequence information was available, including *ACT, GAPDH, EF1α, TEF, TUA, UBC, CYP, RPL3, NAC domain protein and ribosomal protein 18 s*. As Fan and colleagues have recommended *TIP41* and *NTB* as reference genes for the *P. edulis* [42], we obtained the sequence information of these two genes in *D. latiflorus* Munro by homologous clone strategy and added these two genes in our study. Meanwhile, *LAC* and *FER* were selected as the target genes. Confirmation of the specificity of the qRT-PCR primer pairs was conducted through agarose gel electrophoresis and sequencing of the amplicons. The PCR amplification efficiency was estimated, and the reference genes were ranked according to their expression stability across different tissues using three different software packages: geNorm, NormFinder and Bestkeeper. So far, many mathematical softwares have been designed to assess suitable reference genes with the lowest variation and with high stability across different biological samples or under various treatment conditions, among which NormFinder algorithm, GeNorm and BestKeeper are the three most frequently used ones. NormFinder algorithm [45] calculates the overall expression variation of candidate reference gene and produces a stability value which is related to the systematic error of each candidate gene. GeNorm software [24] estimates the gene stability measure (M value) and the candidate gene with the highest M value is regarded as the the most stable reference gene. BestKeeper is a Microsoft Excel-based tool whichcalculates pair-wise correlations [46]. The combined application of the above three softwares would help to identify more reliable reference genes.

We first utilized geNorm software which has been regarded as one of the most authentic methods to identify appropriate reference genes [32], [55], [60], [61]. The geNorm analysis indicated that *EF1α* and *GAPDH* are the two most stable genes across ten tissues or considering all the samples as compared with the other selected genes. *RPL3* were regarded as the most reliable genes when analyzing the samples from seedlings and anther-regenerated plants of different ploidy. Data analysis of expression stability (M value) and normalisation factor variation (Vn/Vn+1) determined that *TEF* and *RPL3* could be added in combination with *GAPDH*/*EF1α* to calculate a normalisation factor based on multiple reference genes when considering ten tissues or all samples [24]. For the reason that geNorm software determines the stability of a candidate gene by pairwise comparison, hence NormFinder and Bestkeeper were used to avoid co-regulation and further assess the analysis results obtained by geNorm [67], [68]. In the case of ten tissues or all samples, both NormFinder and BestKeeper identified *GAPDH* and *EF1α* as the two most stable genes, which supported the geNorm analysis in this experiment. *TEF* and *RPL3* were also among the top-ranked reference genes. When evaluating the samples from seedlings and anther-regenerated plants of different ploidy, *RPL3* was recommended as the first choice, which further verified the geNorm analysis. Synthesizing all the analysis of our datasets, it could be found that different reference genes were recommended to be used in different experimental sets. This result emphasizes the importance of reference genes validation for each experimental condition, especially when samples belong to very different sets, which is consistent with numerous studies by others [34], [35], [40].

*GAPDH* is one of the most commonly used reference genes to normalize gene expression data in qRT-PCR assays. In our study, *GAPDH* exhibited the relatively low level of Ct variation across the samples and was validated as the ideal internal gene for normalization. Studies on validation of reference genes for chickpea [69] and sugarcane [70] have implied that *GAPDH* maintained constant expression in total developmental stages, whereas other studies have shown that *GAPDH* is not stable among different tissues or under diverse environmental conditions [23], [61]. Chang and colleagues found that *GAPDH* was the most stably expressed reference gene in different ages, but exhibited less stable expression in different tissues of *Platycladus orientalis* [41]. For Moso bamboo with closely physiological relationship with Ma bamboo, Fan and colleagues [42] indicated that the traditional reference genes such as *ACT*, *GAPDH, EF1α* and *TUA* which act as housekeeping genes and are widely used in many research fields as reference gene were not suitable reference genes in Moso bamboo. These results indicate that there are no universal reference genes for all plant species, or different developmental stages of one species. Validation of any selected housekeeping gene used as reference gene is thus of great necessity for the acquirements of more accurate and biologically meaningful results in gene expression analysis.

Our data demonstrated that *EF1α* was also ranked in top positions in all samples of *D. latiflorus* Munro based on the results from the three software packages. It has been reported that *EF1α* exhibited stable expression during biotic and abiotic stress in both potato and rice [32], [35]. Czechowski and colleagues compared traditional and novel reference genes in *Arabidopsis* and found that *EF1α* was never represented in the top 100 most stably expressed genes [19]. Even in Moso bamboo, *EF1α* was not recommended as suitable reference gene [42]. This further confirms that it is essential to screen for multiple reference genes for specific species, even each tissue type and stress condition.

*RPL3* was indicated to have optimal performance when analyzing the samples from seedlings and anther-regenerated plants of different ploidy. Ribosomal genes are considered to be good housekeeping genes due to the reason that they are expressed in all cell types to conduct biogenesis of new ribosomes [71.]. Van Raay and colleagues have reported that *RPL3* was expressed ubiquitously in all tissues [72]. During previous study, we have established an efficient plant-regeneration system for *D. latiflorus* Munro by anther culture and obtained anther-regenerated plants of different ploidy [73]. During their growth and development, they exhibit diverse characteristics and the differential genes responsible for this phenomenon are of interest. Therefore, the assessment of a reliable reference gene is important during the process of identifying and quantifying differential expression genes.

Numerous cellular maintenance genes have been selected as reference genes and they are mostly indispensable members taking parts in basic and ubiquitous cellular processes such as glycolytic pathway, protein folding and degradation, components of the cytoskeleton, synthesis of ribosome subunits. However, some traditional housekeeping genes like *ACT* and *TUA* were not found to be expressed stably across the ten tissues of *D. latiflorus* Munro tested in the current study. Recently, several other studies have also discovered that *ACT* could not serve as reliable internal gene for normalization in rice [27], potato [35], *Arabidopsis* [74] and peach [21]. The instability of these commonly used housekeeping genes may be due to the versatility of their functions because they not only perform their basic cellular metabolic functions but also are involved in many other cellular processes. Therefore, the varied expression profile of *ACT* and *TUA* may be attributed to its participation in cell division, cytoplasmic streaming, and the distribution of the plasma membrane proteins other than being a major component of eukaryotic cytoplasmic microfilaments [52].

Although Fan and colleagues have recommended *TIP41* and *NTB* as reference genes for *P. edulis* [42], our analysis showed that they performed poorly and may not be suitable for *D. latiflorus* Munro. This may be because these two bamboos represent two different categories, *P. edulis* belonging to running bamboos (Monopodial) while *D. latiflorus* Munro belonging to clumping bamboos (Sympodial). The discrepancy of the tested samples and different natural surroundings would also exert impacts on the results. These results further confirmed the need to evaluate reference genes for a specific species.

Based on the results from geNorm and Normfinder analysis, *CYP* was characterized as the least stable gene and it was ranked in the bottom position according to the Bestkeeper software. Similarly, some earlier analyses have also showed that *CYP* was not appropriate to be used as the ideal reference genes [35], [75]. The instability of *CYP* expression may be due to the reason that the profile of *CYP* expression is significantly regulated by various internal and external factors such as plant growth or exposure to certain stress inducers [76]. Other reference genes, like *TUA*, *UBC*, and *NAC* displayed variable expression profiles, limiting their application as internal controls. Taken together, these results suggested that a reference gene with stable expression in one species may not be suitable to normalize gene expression for another species, that is to say, reliable reference genes are highly specific for a particular species, thus requiring a careful evaluation for every species.

In summary, this is the first study aimed at validating candidate reference genes for the quantification of transcript levels among various tissues of *D. latiflorus* Munro. In order to get the most reliable results in gene profiling studies, more than one reference gene were recommended as internal controls for relative gene quantification. Our data showed that expression stability varied considerably among genes in different tissue samples. Based on the analysis by the software applications BestKeeper, geNorm and NormFinder, *GAPDH* and *EF1α* were recommended to be the two most suitable reference genes followed by *TEF* and *RPL3* across diverse tissue samples or in all sample pools, while *CYP*, *TUA* and *NAC* were considered to be unsuitable as internal controls. *RPL3* was the best candidate reference gene for samples from seedlings and anther-regenerated plants of different ploidy. *TIP41* and *NTB*, the two ideal internal genes for Moso bamboo, performed poorly in our analysis and may be not good choice for *D. latiflorus* Munro. To validate the feasibility of the reference genes selected in this study, the expression profiles of *LAC* and *FER* were assessed in different experimental sets. The results showed that normalizations using the most stable reference genes (*GAPDH*, *EF1α*, *TEF* and *RPL3*) were coincident and similar to each other, but the normalization was obscured when the least stable reference gene (*CYP*) were used. The transcriptome database of *D. latiflorus* Munro constitutes a new valuable resource for genomic studies and the validation of suitable reference genes would contribute to the future works on genes especially those predicted to be unique for *D. latiflorus* Munro using qRT-PCR.

## Materials and Methods

### Ethics Statement

All necessary permits were obtained for the described field studies. The authority responsible for the bamboo garden is Nanjing Forestry Bureau which provides permissions to collect the samples for our scientific research.

### Plant Materials

*D. latiflorus* Munro was obtained from Nanjing bamboo garden, Fujian Province. Seeds, flowers and tissues including leaves, stem, and root were dissected from four-year-old bamboos and immediately frozen and stored in liquid nitrogen until analysis. The shoots with the height of 15 cm or so were selected to be the representative specimen and the apical, middle and basal parts with the length of 2 cm were sampled respectively. In addition, the leaves of three-year-old anther-regenerated *D. latiflorus* Munro plants of three different ploidy (dodecaploid, hexaploid and triploid) and seedling-derived plants of the same age were also sampled to broaden the experiment scope.

### Total RNA Isolation and First Strand cDNA Synthesis

Total RNAs were extracted from these materials using the Norgan RNA Purification Kit (Norgan Biotek Corp., Ontario, Canada). The quality and quantity of total RNA was analyzed using a NanoDrop2000 spectrophotometer (Thermo, Wilmington, USA) and gel electrophoresis. RNA samples used for cDNA synthesis were picked up according to the criterion that 260/280 wavelength ratio was between 1.8 and 2.1 and 260/230 wavelength ratio was greater than 2.0. The qualified RNA was first applied to the Dnase I step (Invitrogen, Carlsbad, USA) to remove plausible genome DNA contamination, then 3 µg total RNA was used as template for first strand cDNA synthesis using the superscript III first strand synthesis system with 50 µM oligo (dT)_20_ and 50 µM random hexamer priming method according to the protocol of the manufacturer in a final volume of 20 µl. The final cDNA products were diluted 1∶30 with nuclease-free water prior to use in qRT-PCR.

### Primer Design and qRT-PCR

Twelve reference genes except *TIP41* and *NTB* and two target genes were selected from the transcriptome of *D. latiflorus* Munro and gene sequences were deposited in the GenBank (accession numbers are listed in Table 1). The coding sequences for *TIP41* and *NTB* were first amplified by homologous cloning on the basis of the sequence information from Moso bamboo, then sequenced, characterized and submitted to the Genbank. The primers for the twelve reference genes and two target genes were designed using Primer 3 software (http://frodo.wi.mit.edu/primer3/) (Table 2). In order to confirm the primer specificity, all primer pairs were initially tested via standard RT-PCR using the Premix Ex Taq (TaKaRa, Japan) and a single amplification product of the expected size for each gene was verified by electrophoresis on a 2% agarose gel and stained with ethidium bromide. Negative PCR control without templates was also conducted for each primer pair. qRT-PCR reactions were performed in 96-well plates with an Applied Biosystems 7300 Real-Time PCR system using SYBR® Premix Ex Taq™ (TaKaRa, Japan) in a 20 µl reaction volume (containing 2 µl cDNA reaction mixture, 10 µl SYBR® Premix Ex Taq™, 0.4 µl ROX Reference Dye, and 0.4 µl each primer). The thermal profile of the reaction followed the instructions that recommended by the manufacturer (10 s at 95^o^C, 40 cycles of 95^o^C for 5 s, and 60^o^C for 31 s). Melting dissociation curve, obtained by heating the amplicon from 60^o^C to 95^o^C, was carried out to verify the presence of gene-specific peaks and the absence of primer dimers. All qRT-PCR reactions were performed in technical triplicate. The final threshold cycle (Ct) values were the mean of four values.

### Determination of Reference Gene Expression Stability Using geNorm, Normfinder and BestKeeper

The slope of a standard curve serves as an indication of the efficiency of the qRT- PCR. To generate a standard curve, Ct values of 10-fold series dilution of the mixed cDNA template for each primer pair are plotted against the logarithm of input amount of standard material. The correlation coefficients (R^2^) and the slope could be calculated from the resulting standard curve and amplification efficiencies (*E*) could be determined from the given slope generated in Microsoft Excel 2010 according to the equation *E* = (10^−1/slope^−1)×100 [18].

In order to identify a suitable reference gene, the gene expression stability was statistically analyzed based on the results of three different types of Microsoft Excel-based software: geNorm [24], NormFinder [45], and BestKeeper [46]. GeNorm is an algorithm that selects an ideal pair of reference genes out of candidate genes and it relies on the principle that the expression ratio of two ideal reference genes should not be influenced by external factors, regardless of the experiment condition or tissue type. Through the calculation and comparison of the reference gene expression stability measure (M value) of all candidate genes, the gene with highest M-value was eliminated and the process was repeated until only two genes left. Genes with low M values were characterized as the most stable expression gene.

The NormFinder reference tool, another algorithm that attempts to find the optimum reference genes out of a group of candidate reference genes, could calculate the variance within group as well as the variance between groups. According to the analysis, the candidate reference gene expression stability will be estimated and the gene with the lowest stability value will be top ranked.

BestKeeper evaluates the stabilities of candidate reference genes based on the coefficient of correlation to the BestKeeper index, which is the geometric mean of the Ct values of all candidate reference genes [46], [68]. The coefficient of variance (CV) and the standard deviation (SD) of the Ct values are also calculated using the whole data set and all the Ct values are analyzed as a total data set [46]. Reference genes exhibiting the lowest coefficient of variance and standard deviation (CV±SD) are identified as the most stable genes. Genes showing a SD greater than 1 are considered to be unacceptable [77].

The above three software packages were used following the manufacturer’s protocols. For geNorm and NormFinder, the raw Ct values needed to be transformed into the required data input format. The maximum expression level of each gene indicated by the lowest Ct value was used as a control and was set to a value of 1. Relative expression levels were then calculated from Ct values using the formula: 2^−ΔCt^, in which ΔCt = each corresponding Ct value–minimum Ct value. The obtained results were then further analyzed with geNorm and NormFinder. BestKeeper analyses were based on untransformed Ct values.

## References

[1] Wu ZY, Raven PH, Hong DY (2006) Floral of China: Poaceae: Science Press, Beijing, and Missouri Botanical Gardern Press, St Louis.

[2] Barker NP, Clark LG, Davis JI, Duvall MR, Guala GF, et al (2001) Phylogeny and subfamilial classification of the grasses (Poaceae). Ann Missouri Bot Garden 88: 373–457.

[3] Scurlock JMO, Dayton DC, Hames B (2000) Bamboo: an overlooked biomass resource? Biomass Bioenerg 19: 229–244.

[4] Hsu YH, Annamalai AP, Lin CS, Chen YY, Chang WC, et al (2000) A sensitive method for detecting bamboo mosaic virus (BaMV) and establishment of BaMV-free meristem tip cultures. Plant Path 49: 101–107.

[5] Bystriakova N, Kapos V, Stapleton C, Lysenko I (2003) Bamboo biodiversity: information for planning conservation and management in the Asia-Pacific region. UNEP-WCMC Biodiversity Series 14.

[6] Bystriakova N, Kapos V, Stapleton C, Lysenko I (2004) Bamboo biodiversity: Africa, Madgascar and the Americas. UNEP-WCMC Biodiversity Series 19.

[7] Hilton-Taylor C, Mittermeier RA (2000) 2000 IUCN red list of threatened species: IUCN–The World Conservation Union.

[8] Walter KS, Gillett HJ (1998) 1997 IUCN red list of threatened plants: IUCN.

[9] Pilcher HR (2004) Bamboo under extinction threat. Nature doi:10.1038/news040510-2.

[10] Yoo WG, Kim TI, Li S, Kwon OS, Cho PY, et al (2009) Reference genes for quantitative analysis on *Clonorchis sinensis* gene expression by real-time PCR. Parasitol Res 104: 321–328. 18815810 10.1007/s00436-008-1195-x

[11] Ohdan T, Francisco PB, Sawada T, Hirose T, Terao T, et al (2005) Expression profiling of genes involved in starch synthesis in sink and source organs of rice. J Exp Bot 56: 3329–3244. 10.1093/jxb/eri292 16275672

[12] Ishimaru T, Hirose T, Matsuda T, Goto A, Takahashi K, et al (2005) Expression patterns of genes encoding carbohydrate-metabolizing enzymes and their relationship to grain filling in rice (*Oryza sativa* L.): comparsion of caryopses located at different positions in a panicle. Plant Cell Physiol 46: 620–628. 15701658 10.1093/pcp/pci066

[13] Ginzinger DG (2002) Gene quantification using real-time quantitative PCR: An emerging technology hits the mainstream. Exp Hematol 30: 503–512. 12063017 10.1016/s0301-472x(02)00806-8

[14] Mahoney DJ, Carey K, Fu MH, Snow R, Cameron-Smith D, et al (2004) Realtime RT-PCR analysis of housekeeping genes in human skeletal muscle following acute exercise. Physiol Genomics 18: 226–231. 15161965 10.1152/physiolgenomics.00067.2004

[15] Demidenko NV, Logacheva MD, Penin AA (2011) Selection and validation of reference genes for quantitative real-time PCR in Buckwheat (*Fagopyrum esculentum*) based on transcriptome sequence data. PLoS ONE 6: e19434. 21589908 10.1371/journal.pone.0019434 PMC3093374

[16] Hoenemann C, Hohe A (2011) Selection of reference genes for normalization of quantitative real-time PCR in cell cultures of *Cyclamen persicum* . Electron J Biotechnol 14: 12–13.

[17] Everaert BR, Boulet GA, Timmermans JP, Vrints CJ (2011) Importance of suitable reference gene selection for quantitative real-time PCR: special reference to mouse myocardial infarction studies. PLoS ONE 6: e23793. 21858224 10.1371/journal.pone.0023793 PMC3157472

[18] Radonic A, Thulke S, Mackay IM, Landt O, Siegert W, et al (2004) Guideline to reference gene selection for quantitative real-time PCR. Biochem Biophys Res Commun 313: 856–862. 14706621 10.1016/j.bbrc.2003.11.177

[19] Czechowski T, Stitt M, Altmann T, Udvardi MK, Scheible WR (2005) Genome-wide identification and testing of superior reference genes for transcript normalisation in Arabidopsis. Plant physiol 139: 5–17. 16166256 10.1104/pp.105.063743 PMC1203353

[20] Yang LT, Pan AH, Jia JW, Ding JY, Chen JX, et al (2005) Validation of a tomato-specific gene, LAT52, used as an endogenous reference gene in qualitative and real-time quantitative PCR detection of transgenic tomatoes. J Agric Food Chem 53: 183–190. 15656646 10.1021/jf0493730

[21] Tong Z, Gao Z, Wang F, Zhou J, Zhang Z (2009) Selection of reliable reference genes for gene expression studies in peach using realtime PCR. BMC Mol Biol 10: 71–82. 19619301 10.1186/1471-2199-10-71 PMC3224724

[22] Kwon MJ, Oh E, Lee S, Roh MR, Kim SE, et al (2009) Identification of novel reference genes using multiplatform expression data and their validation for quantitative gene expression analysis. PLoS ONE 4: e6162. 19584937 10.1371/journal.pone.0006162 PMC2703796

[23] Løvdal T, Lillo C (2009) Reference gene selection for quantitative real-time PCR normalization in tomato subjected to nitrogen, cold, and light stress. Anal Biochem 387: 238–242. 19454243 10.1016/j.ab.2009.01.024

[24] Vandesompele J, De Preter K, Pattyn F, Poppe B, Van Roy N, et al (2002) Accurate normalization of real-time quantitative RT-PCR data by geometric averaging of multiple internal control genes. Genome Biol 3: RESEARCH0034. 12184808 10.1186/gb-2002-3-7-research0034 PMC126239

[25] Tricarico C, Pinzani P, Bianchi S, Paglierani M, Distante V, et al (2002) Quantitative real-time reverse transcription polymerase chain reaction: normalization to rRNA or single housekeeping genes is inappropriate for human tissue biopsies. Anal Biochem 309: 293–300. 12413463 10.1016/s0003-2697(02)00311-1

[26] Remans T, Smeets K, Opdenakker K, Mathijsen D, Vangronsveld J, et al (2008) Normalisation of real-time RT-PCR gene expression measurements in Arabidopsis thaliana exposed to increased metal concentrations. Planta 227: 1343–1349. 18273637 10.1007/s00425-008-0706-4

[27] Jain M, Nijhawan A, Tyagi AK, Khurana JP (2006) Validation of housekeeping genes as internal control for studying gene expression in rice by quantitative realtime PCR. Biochem Biophys Res Commun 345: 646–651. 16690022 10.1016/j.bbrc.2006.04.140

[28] Kim BR, Nam HY, Kim SU, Kim SI, Chang YJ (2003) Normalization of reverse transcription quantitative-PCR with housekeeping genes in rice. Biotechnol Lett 25: 1869–1872. 14677714 10.1023/a:1026298032009

[29] Hong SY, Seo PJ, Yang MS, Xiang F, Park CM (2008) Exploring valid reference genes for gene expression studies in Brachypodium distachyon by real-time PCR. BMC Plant Biol 8: 112. 18992143 10.1186/1471-2229-8-112 PMC2588586

[30] Paolacci AR, Tanzarella OA, Porceddu E, Ciaffi M (2009) Identification and validation of reference genes for quantitative RT-PCR normalization in wheat. BMC Mol Biol 10: 11. 19232096 10.1186/1471-2199-10-11 PMC2667184

[31] Faccioli P, Ciceri GP, Provero P, Stanca AM, Morcia C, et al (2007) A combined strategy of ‘‘in silico’’ transcriptome analysis and web search engine optimization allows an agile identification of reference genes suitable for normalization in gene expression studies. Plant Mol Biol 63: 679–688. 17143578 10.1007/s11103-006-9116-9

[32] Jian B, Liu B, Bi YR, Hou WS, Wu CX, et al (2008) Validation of internal control for gene expression study in soybean by quantitative real-time PCR. BMC Mol Biol 9: 59. 18573215 10.1186/1471-2199-9-59 PMC2443375

[33] Libault M, Thibivilliers S, Bilgin D, Radwan O, Benitez M, et al (2008) Identification of four soybean reference genes for gene expression normalization. Plant Genome 1: 44–54.

[34] Expósito-Rodríguez M, Borges AA, Borges-Pérez A, Pérez JA (2008) Selection of internal control genes for quantitative real-time RT-PCR studies during tomato development process. BMC Plant Biol 8: 131. 19102748 10.1186/1471-2229-8-131 PMC2629474

[35] Nicot N, Hausman JF, Hoffmann L, Evers D (2005) Housekeeping gene selection for real-time RT-PCR normalization in potato during biotic and abiotic stress. J Exp Bot 56: 2907–2914. 16188960 10.1093/jxb/eri285

[36] Iskandar HM, Simpson RS, Casu RE, Bonnett GD, Maclean DJ (2004) Comparison of reference genes for quantitative real-time polymerase chain reaction analysis of gene expression in sugarcane. Plant Mol Biol 22: 325–337.

[37] Brunner AM, Yakovlev IA, Strauss SH (2004) Validating internal controls for quantitative plant gene expression studies. BMC Plant Biol 4: 14. 15317655 10.1186/1471-2229-4-14 PMC515301

[38] Peng ZH, Lu TT, Li LB, Liu XH, Gao ZM, et al (2010) Genome-wide characterization of the biggest grass, bamboo, based on 10,608 putative full-length cDNA sequences. BMC plant biology 10: 116–128. 20565830 10.1186/1471-2229-10-116 PMC3017805

[39] Liu M, Qiao G, Jiang J, Yang H, Xie L, et al (2012) Transcriptome Sequencing and *De Novo* Analysis for Ma Bamboo (*Dendrocalamus latiflorus* Munro) Using the Illumina Platform. PLoS ONE 7: e46766. 23056442 10.1371/journal.pone.0046766 PMC3463524

[40] Narsai R, Ivanova A, Ng S, Whelan J (2010) Define reference genes in *Oryza sativa* using organ, development, biotic and abiotic transcriptome datasets. BMC Plant Biology 10: 56. 20353606 10.1186/1471-2229-10-56 PMC2923530

[41] Chang E, Shi S, Liu J, Cheng T, Xue L, et al (2012) Selection of Reference Genes for Quantitative Gene Expression Studies in *Platycladus orientalis* (Cupressaceae) Using Real-Time PCR. PLoS ONE 7: e33278. 22479379 10.1371/journal.pone.0033278 PMC3316566

[42] Fan C, Ma J, Guo Q, Li X, Wang H, et al (2013) Selection of Reference Genes for Quantitative Real-Time PCR in Bamboo (*Phyllostachys edulis*). PLoS ONE 8: e56573. 23437174 10.1371/journal.pone.0056573 PMC3577859

[43] Scharlaken B, de Graaf DC, Goossens K, Brunain M, Peelman LJ, et al (2008) Reference gene selection for insect expression studies using quantitative realtime PCR: The head of the honeybee, *Apis mellifera*, after a bacterial challenge. J Insect Sci 8: 1–10.

[44] Jacob F, Guertler R, Naim S, Nixdorf S, Fedier A, et al (2013) Careful selection of reference genes is required for reliable performance of RT-qPCR in human normal and cancer cell lines. PLoS One 8: e59180. 23554992 10.1371/journal.pone.0059180 PMC3598660

[45] Andersen CL, Jensen JL, Orntoft TF (2004) Normalization of real-time quantitative reverse transcription-PCR data: a model-based variance estimation approach to identify genes suited for normalization, applied to bladder and colon cancer data sets. Cancer Res 64: 5245–5250. 15289330 10.1158/0008-5472.CAN-04-0496

[46] Pfaffl MW, Tichopad A, Prgomet C, Neuvians TP (2004) Determination of stable housekeeping genes, differentially regulated target genes and sample integrity: BestKeeper–Excel-based tool using pair-wise correlations. Biotechnol Lett 26: 509–515. 15127793 10.1023/b:bile.0000019559.84305.47

[47] Briat JF, Ravet K, Arnaud N, Duc C, Boucherez J, et al (2010) New insights into ferritin synthesis and function highlight a link between iron homeostasis and oxidative stress in plants. Ann Bot 105: 811–822. 19482877 10.1093/aob/mcp128 PMC2859905

[48] Gachon C, Mingam A, Charrier B (2004) Real-time PCR: what relevance to plant studies? J Exp Bot 55: 1445–1454. 15208338 10.1093/jxb/erh181

[49] Wong ML, Medrano JF (2005) Real-time PCR for mRNA quantitation. BioTechniques 39: 75–85. 16060372 10.2144/05391RV01

[50] Nolan T, Hands RE, Bustin SA (2006a) Quantification of mRNA using real-time RT-PCR. Nat Protocols 1: 1559–1582. 17406449 10.1038/nprot.2006.236

[51] Dean JD, Goodwin PH, Hsiang T (2002) Comparison of relative RT-PCR and northern blot analyses to measure expression of β-1, 3-glucanase in *Nicotiana benthamiana* infected with *Colletotrichum destructivum* . Plant Mol Biol Rep 20: 347–356.

[52] Stürzenbaum SR, Kille P (2001) Control genes in quantitative molecular biological techniques: the variability of invariance. Comp Biochem Physiol B Biochem Mol Biol 130: 281–289. 11567890 10.1016/s1096-4959(01)00440-7

[53] Bezier A, Lambert B, Baillieul F (2002) Study of defense-related gene expression in grapevine leaves and berries infected with *Botrytis cinerea* . Eur J Plant Pathol 108: 111–120.

[54] Thomas C, Meyer D, Wolff M, Himber C, Alioua M, et al (2003) Molecular characterization and spatial expression of the sunflower *ABP1* gene. Plant Mol Biol 52: 1025–1036. 14558662 10.1023/a:1025482432486

[55] Gutierrez L, Mauriat M, Pelloux J, Bellini C, Van Wuytswinkel O (2008) Towards a systematic validation of references in real-time RT–PCR. Plant Cell 20: 1734–1735. 18664615 10.1105/tpc.108.059774 PMC2518241

[56] Guénin S, Mauriat M, Pelloux J, Van Wuytswinkel O, Bellini C, et al (2009) Normalization of qRT–PCR data: the necessity of adopting a systematic, experimental conditions-specific, validation of references. J Exp Bot 60: 487–493. 19264760 10.1093/jxb/ern305

[57] Hruz T, Wyss M, Docquier M, Pfaffl MW, Masanetz S, et al (2011) RefGenes: identification of reliable and condition specific reference genes for RT–qPCR data normalization. BMC Genomics 12: 156. 21418615 10.1186/1471-2164-12-156 PMC3072958

[58] Podevin N, Krauss A, Henry I, Swennen R, Remy S (2012) Selection and validation of reference genes for quantitative RT-PCR expression studies of the non-model crop Musa. Mol Breed 30: 1237–1252. 23024595 10.1007/s11032-012-9711-1 PMC3460175

[59] Miranda Vde J, Coelho RR, Viana AA, de Oliveira Neto OB, Carneiro RM, et al (2013) Validation of reference genes aiming accurate normalization of qPCR data in soybean upon nematode parasitism and insect attack. BMC Res Notes 6: 196. 23668315 10.1186/1756-0500-6-196 PMC3660166

[60] Mukesh J, Nijhawan A, Tyagi AK, Khurana JP (2006) Validation of housekeeping genes as internal control for studying gene expression in rice by quantitative real-time PCR. Biochem Biophys Res Commun 345: 646–651. 16690022 10.1016/j.bbrc.2006.04.140

[61] Marino ER, Borges AA, Perez AB, Perez JA (2008) Selection of internal control genes for quantitative real-time RT-PCR studies during tomato development process. BMC Plant Biol 8: 131. 19102748 10.1186/1471-2229-8-131 PMC2629474

[62] De Keyser E, Desmet L, Van Bockstaele E, De Riek J (2013) How to perform RT-qPCR accurately in plant species? A case study on flower colour gene expression in an azalea (*Rhododendron simsii* hybrids) mapping population. BMC Mol Biol 14: 13. 23800303 10.1186/1471-2199-14-13 PMC3698002

[63] Tian B, Chen Y, Yan Y, Li D (2005) Isolation and ectopic expression of a bamboo MADS-box gene. Chin Sci Bull 50: 145–151.

[64] Tian B, Chen Y, Li D, Yan Y (2006) Cloning and characterization of a bamboo Leafy Hull Sterile1 homologous gene. DNA Sequence 17: 143–151. 17076257 10.1080/10425170600699877

[65] Xu H, Chen LJ, Qu LJ, Gu HY, Li DZ (2010) Functional conservation of the plant EMBRYONIC FLOWER2 gene between bamboo and *Arabidopsis* . Biotechnol Lett 32: 1961–1968. 20676919 10.1007/s10529-010-0362-1

[66] Ahi EP, Guðbrandsson J, Kapralova KH, Franzdóttir SR, Snorrason SS, et al (2013) Validation of Reference Genes for Expression Studies during Craniofacial Development in Arctic Charr. PLoS ONE 8: e66389. 23785496 10.1371/journal.pone.0066389 PMC3681766

[67] Cruz F, Kalaoun S, Nobile P, Colombo C, Almeida J, et al (2009) Evaluation of coffee reference genes for relative expression studies by quantitative real-time RT-PCR. Mol Breed 23: 07–616.

[68] Zhao WJ, Li Y, Gao PF, Sun ZH, Sun TS, et al (2011) Validation of reference genes for real-time quantitative PCR studies in gene expression levels of *Lactobacillus casei* Zhang. J Ind Microbiol Biotechnol 38: 1279–1286. 21104423 10.1007/s10295-010-0906-3

[69] Garg R, Sahoo A, Tyagi AK, Jain M (2010) Validation of internal control genes for quantitative gene expression studies in chickpea. Biochem Biophys Res Commun 396: 283–288. 20399753 10.1016/j.bbrc.2010.04.079

[70] Long X, Wang JR, Ouellet T, Rocheleau H, Wei Y, et al (2010) Genome-wide identification and evaluation of novel internal control genes for Q-PCR based transcript normalization in wheat. Plant Mol Bio 74: 307–311. 20658259 10.1007/s11103-010-9666-8

[71] Hsiao LL, Dangond F, Yoshida T, Hong R, Jensen RV, et al (2001) A compendium of gene expression in normal human tissues. Physiol Genomics 7: 97–104. 11773596 10.1152/physiolgenomics.00040.2001

[72] Van Raay TJ, Connors TD, Klinger KW, Landes GM, Burn TC (1996) A novel ribosomal protein L3-like gene (RPL3L) maps to the autosomal dominant polycystic kidney disease gene region. Genomics 37: 172–176. 8921388 10.1006/geno.1996.0538

[73] Qiao GR, Li HY, Liu MY, Jiang J, Yin YF, et al (2013) Callus induction and plant regeneration from anthers of *Dendrocalamus latiflorus* Munro. In Vitro Cell Dev Biol–Plant 49: 375–382.

[74] Gutierrez L, Mauriat M, Guenin S, Pelloux J, Lefebvre JF, et al (2008a) The lack of a systematic validation of reference genes: a serious pitfall undervalued in reverse transcription-polymerase chain reaction (RT-PCR) analysis in plants. Plant Biotechnol J 6: 609–618. 18433420 10.1111/j.1467-7652.2008.00346.x

[75] Reid KE, Olsson N, Schlosser J, Peng F, Lund ST (2006) An optimized grapevine RNA isolation procedure and statistical determination of reference genes for real-time RT-PCR during berry development. BMC Plant Biol 6: 27. 17105665 10.1186/1471-2229-6-27 PMC1654153

[76] Marivet J, Frendo P, Burkard G (1992) Effects of antibiotic stresses on cyclophilin in maize and bean and sequence analysis of bean cyclophilin cDNA. Plant Sci 84: 171–178.

[77] Migocka M, Papierniak A (2010) Identification of suitable reference genes for studying gene expression in cucumber plants subjected to abiotic stress and growth regulators. Mol Breeding 28: 343–357.

